# A new duck circovirus sequence, detected in velvet scoter (*Melanitta fusca*) supports great diversity among this species of virus

**DOI:** 10.1186/s12985-015-0352-y

**Published:** 2015-08-08

**Authors:** Anna Karolina Matczuk, Marta Krawiec, Alina Wieliczko

**Affiliations:** Department of Pathology, Division of Microbiology, The Faculty of Veterinary Medicine, Wrocław University of Environmental and Life Sciences, Norwida 31, 50-375 Wrocław, Poland; Department of Epizootiology and Clinic of Bird and Exotic Animals, The Faculty of Veterinary Medicine, Wrocław University of Environmental and Life Sciences, Pl. Grunwaldzki 45, 50-366 Wrocław, Poland

## Abstract

**Background:**

The aim of this study was to investigate the presence of circoviruses in wild bird populations, in Poland. Circoviruses possess immuno-suppressive properties and might interfere with the health of wild birds.

**Method:**

83 birds, which belonged to 23 species, were tested with broad-range, nested PCR. The obtained PCR products were sequenced and new primers designed, to analyse the full-length, viral genome. A phylogenetic analysis was conducted, to find any relationship to known circoviruses.

**Results:**

The circovirus DNA sequence was found in 4 birds. All samples originated from the velvet scoter (*Melanitta fusca*) a marine duck from the *Merginae* sub-family. Birds which tested positive for the circovirus were found dead in fishing nets, off the Baltic coast. During post-mortem examination, carcasses of two of the scoters showed only light emaciation, while the two other birds appeared healthy. The obtained, full-length, circovirus sequence revealed 1,988 nucleotides and the presence of typical features (i.e. Cap, Rep and ORF3). Nucleotide similarity to other duck circoviruses was 84 to 86 %. Phylogenetic analysis of the complete genome and cap gene, indicated that the new circovirus is related to known duck circoviruses, especially to sub-types sometimes referred to as duck circovirus genotype 1, but not genotype 2.

**Conclusions:**

In this study, we have reported a new duck circovirus sequence detected in the velvet scoter, a species of marine duck. Sequence comparison and phylogenetic analysis of the new virus sequence support previous reports that duck circovirus (DuCV) is a species with a high degree of diversity. The viral sequence obtained from the velvet scoter suggests that DuCV may infect birds from the *Anatinae* sub-family. More studies are needed to prove if the velvet scoter and other marine ducks act as a reservoir for DuCV.

## Background

Viruses from the *Circoviridae* family infect a wide range of vertebrates. The virions are about 20 nm in diameter and are composed of naked nucleocapsids, with a circular, single-stranded DNA (ssDNA) genome of approximately 2 kbp, in size [1]. The genome organization is ambisense, meaning that the two main, open reading frames (ORFs) responsible for encoding replicase (Rep) and capsid (Cap) respectively, are arranged inversely [1]. Some circoviruses also encode the third, small protein, known as ORF3, which is usually inversely nested, within the coding sequence of Rep. Between the stop codons of the ORFs of Cap and Rep, a hairpin -like structure is known to consist of a palindromic sequence. This structure is the point of origin for replication (Ori) of the circovirus [2].

In domestic animals, circovirus infections might be sub-clinical, but often manifest themselves as serious diseases, with a high, economic impact [3]. Some examples include, porcine circovirus 2 (PCV2) affecting pigs worldwide [4], psittacine beak and feather disease virus (PBFDV) which is a causative agent of the aforementioned disease, in many psittacine birds [5]. In chickens, the chicken anaemia virus (CAV) which belongs to the Gyrovirus genus, is responsible for the depletion of immune cells, immuno-suppression and growth abnormalities [6]. In domestic ducks, duck circovirus (DuCV) affects mostly young animals. The disease is associated with feathering disorders, poor body condition, low body weight and severe immuno-suppression [7]. The International Committee on the Taxonomy of Viruses (ICTV) recognises DuCV as a single species. However, based on the genomic sequences of DuCV, some scientists divide this circovirus into two genotypes; DuCV 1 and DuCV 2 [8, 9]. A similar disease occurs in domestic geese and is caused by the closely related goose circovirus (GoCV) [10].

In wild birds, different circovirus species have been detected and characterized, among them, viruses infecting finch, gull, raven and starling [11–13]. In wild waterfowl, two different types of swan circovirus have been discovered in mute swans [14]. Due to minimal data on circovirus infections, among the free-roaming, avian species available in the world, it is supposed that circovirus infections, in wild birds, display similar symptoms to those observed in domestic birds. In some wild bird species, feather abnormalities have been observed, but primarily, circovirus infections are asymptomatic and associated with immuno-suppression, leading to bird emaciation and an increased susceptibility to secondary pathogens [12, 15, 16].

Cross-infection between closely related avian species remains a possibility, but only between related species, because of a high degree of circovirus host-specificity. Cross-infection between free-living ducks or geese and their farmed counterparts are also considered possible [16].

Although it is known that circovirus infections of farmed geese and ducks are common and widespread, at present, there is a lack of information about the prevalence of the duck or goose circovirus and about the affinity of free-living, bird circoviruses to those isolated from domestic birds.

The aim of this study was to investigate the presence of circoviruses in the wild bird population of Poland. The new DuCV DNA sequence was detected in one of the species: the velvet scoter (*Melanitta fusca*) and subsequently characterized.

## Results

**Fig. 1:**
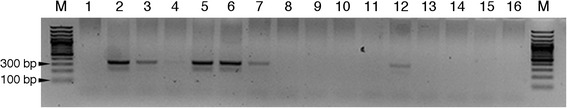
Agarose gel (2 %) picture of the nested PCR amplification of the circovirus. M: molecular marker, 1-negative control, 2-PBFD positive control, 3–4 velvet scoter 23, 5–6 velvet scoter 1, 7–8 velvet scoter 3, 9–10 velvet scoter 6, 11–12 velvet scoter 8, 13–14 great cormorant 1, 15–16 great cormorant 23. Samples: 3,5,7,9,11,13,15 originated from the spleen, samples 4,6,8,10,12,14,16 originated from the liver. Bp: base pair

In broad-range, nested PCR, amplicons of the required size (about 350 bp) were obtained from 4 out of the 83 birds tested (with a prevalence of 4.2 %) (Fig. 1, Table 1). All positive samples originated from the velvet scoter (*Melanitta fusca*) an endangered species of migratory, marine duck from the order, *Anseriformes* [17]. During necropsy, no significant lesions were found, but two circovirus positive birds showed only light emaciation, with slight muscle atrophy.

**Table 1:** DNA of circovirus detected in free-living birds

Order of birds	Species of bird (english/latin)	Number of birds tested	Number (%) of circovirus positive birds
*Anseriformes*	mallard duck/*Anas platyrhynchos*	6	0 (0)
	long-tailed duck/*Clangula hyemalis*	1	0 (0)
	velvet scoter/*Melanitta fusca*	23	4 (17.4)
	common scoter/*Melanitta nigra*	1	0 (0)
*Apodiforme*s	common swift/*Apus apus*	1	0 (0)
*Charadriformes*	razorbill/*Alca torda*	1	0 (0)
	guillemot/*Uriaa alge*	1	0 (0)
*Falconiformes*	kestrel/*Flaco tinnunculus*	1	0 (0)
*Gaviformes*	red-throated/*Gavia stellata*	1	0 (0)
*Gruiformes*	eurasian coot/*Fulica atra*	6	0 (0)
*Passerifomes*	eurasian siskin/*Carduelis spinus*	6	0 (0)
	green finch/*Carduelis chloris*	4	0 (0)
	rook/*Corvus frugilegus*	1	0 (0)
	blackbird/*Turdus melura*	1	0 (0)
	magpie/*Pica pica*	1	0 (0)
	black redstart/*Phoenicurus ochruros*	1	0 (0)
	hooded crow/Corvus cornix	1	0 (0)
*Podicipediformes*	great crested grebe/*Podiceps cristatus*	2	0 (0)
*Strigiformes*	little owl/*Athene noctua*	1	0 (0)
	snowy owl/*Bubo scandiacus*	1	0 (0)
	pygmy owl/*Glausidium passerinum*	1	0 (0)
	tawny owl/*Stric aluco*	2	0 (0)
Suliformes	great cormorant/*Phalacrocorax carbo*	18	0 (0)
In total		83	4 (4.82)

In total, 23 velvet scoters were tested, which means that prevalence in this bird species was 17.4 %. In some birds, the virus was detected only in the DNA sample from the liver (bird 8) the spleen (bird 3) or, in both samples (bird 23 and bird 1) (Fig. 1). A smaller, weaker PCR product was also visible on the gel of positive samples (Fig. 1). Circovirus DNA was not detected in any other tested bird species. The 350 bp PCR products were sequenced and analysed with BLAST, which showed that these partial sequences of the *rep* gene have a nucleic acid similarity of 88 to 91 %, with those of known duck circoviruses. Using primers specific to duck circovirus, the 600 bp product corresponding to a partial, *rep* gene sequence was obtained from all 4 positive samples, in nested PCR, while the DNA from the other 19 velvet scoters was negative. BLAST analysis of the amplified 600 bp product, showed that these partial sequences of the *rep* gene have a nucleic acid similarity of 91 to 92 %, with those of known duck circoviruses, and 99 % among themselves. Amplification of the 1,700 bp product, comprising of the remaining circovirus sequence, was possible for 3 out of 4 samples (sample 1, 3, and 8, but not 23). These PCR products were subjected to cloning (using a PCR cloning kit, Qiagen). However, regardless of multiple attempts and protocol optimizations, we were unable to obtain any positive clone. Direct sequencing, with multiple primers, revealed the whole, circular, ssDNA circovirus sequence of 1,988 nt, which was deposited in GenBank (accession number KP943594). In this paper, the new viral sequence will be called DuCV VS.

BLAST analysis showed that the whole genome sequence of DuCV VS is most similar (86 % similarity) to the DuCV strain HZ09 (EU344802.1).

**Fig. 2:**
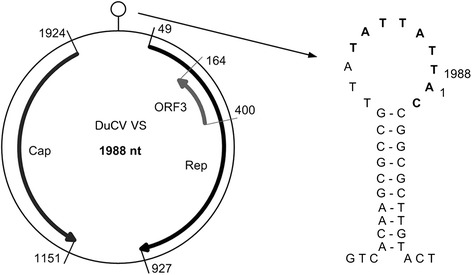
Visualisation of duck circovirus genome isolated from velvet scoter. Nucleotide position indicated for each ORF. Rep - replicase, Cap - capsid protein, ORF3 open reading frame 3. Hairpin like palindromic structure, the origin of replication is shown on the right

The new circovirus sequence has the typical structure of the circovirus; a 9-mer palindromic structure, located in the putative loop of the hairpin and a stem of 10 complementary base pairs (Fig. 2). Three ORFs, *rep*, *cap* and ORF3 were predicted and are depicted in Fig. 2. The predicted Rep protein sequence consists of 292 amino acids and share an amino acid similarity of 91 to 96 %, to other duck circoviruses, and 81 to 83 % to goose circoviruses (BLASTp). The predicted Cap protein consists of 257 amino acids and has an amino acid similarity of 91 % among other duck circoviruses (Emboss Needle) and 47 % to goose circovirus (BLASTp). The predicted ORF3 protein consists of 73 amino acids.

**Fig. 3:**
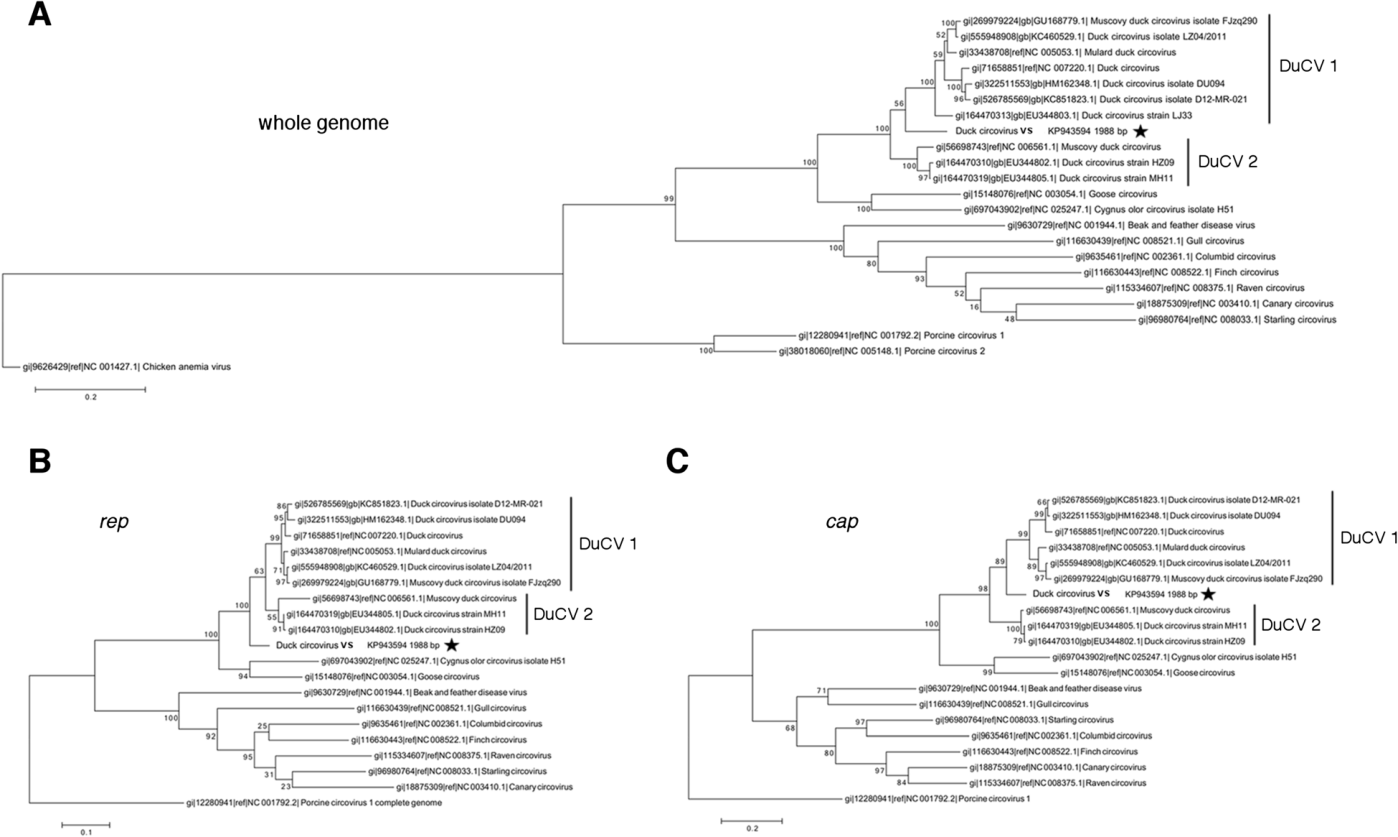
Phylogenetic analysis of new DuCV circovirus sequence. Phylogenetic analysis of circovirus sequences obtained, using the maximum likelihood method, with bootstrap 1,000 (Mega 6.06). **a**: analysis of whole genome sequence; **b**: analysis of *rep* gene sequence; **c**: analysis of *cap* gene sequence. DuCV 1- duck circovirus genotype 1, DuCV 2- duck circovirus genotype 2, VS- velvet scoter, *rep* - replicase gene, *cap* - capside gene. The sequence of the circovirus isolated from the velvet scoter is marked with a star. Other sequences were obtained from GeneBank, accession numbers of those sequences are included in the phylogenetic tree. The DuCV genotypes 1 and 2 are not recognised by ICTV, but are annotated here, for clarity of the figure

A phylogenetic tree was constructed, based on the alignment of whole genome sequences of 9 duck circovirus strains, other bird circoviruses, as well as porcine circoviruses PCV1 and PCV2. The tree was rooted to the Gyrovirus CAV. The duck circovirus sequences chosen for analysis were either reference sequences, or representative strains, for known genotypes [9]. The analysis of whole genome sequences and *cap* gene sequences showed that DuCV VS clusters together, with duck circoviruses of genotype 1 (DuCV 1) (Fig. 3a and c). However, when only the phylogenetic relationship of the *rep* gene was analyzed, the DuCV VS clustered outside the DuCV of both genotypes 1 and 2 (Fig. 3b). Results were the same, regardless of which phylogenetic method was used for the analysis (only the result of the maximum likelihood method is presented). Amino acid alignment of the Rep protein showed that there are 15 and 21 amino acid substitutions compared to Rep, from DuCV 2 (GenBank NC 006561) and from DuCV 1 (GeneBank NC 007220.1) respectively. Amino acid alignment of the Cap protein showed 20 and 25 amino acids substitution between the velvet scoter isolate and the aforementioned DuCV 1 and DuCV 2, respectively.

## Discussion

In this study, we have reported the discovery of a new, circular ssDNA genomic sequence from the liver and spleen samples of a marine duck, the velvet scoter, as collected in Poland.

The circovirus infection of *Anseriformes* is multi-systemic and the virus can be detected in the bursa of Fabricius, spleen, liver, kidney and thymus [18, 10]. The bursa is the most predominant place for circovirus replication, in birds. Unfortunately, the preparation of this organ is possible only in young birds. In other visceral organs, circovirus DNA can be detected on various days, post-infection. In the case of the experimental GoCV infection of young geese, viral DNA was detected in liver only 28 days post-infection (d.p.i.) and in the spleen 49 d.p.i., while in the bursa of Fabricius, at all tested time points, 28, 35 and 49 d.p.i. [10]. In our study, examined birds showed the presence of viral DNA, in both liver and spleen, or only in one organ of the two tested.

Only 2 of the circovirus positive birds showed slight emaciation, so it seems that the infection is only sub-clinical, in adult velvet scoter. To confirm this, more samples should be analysed, especially ones originating from young birds. This is also important, because the velvet scoter is an endangered species [17].

Interestingly, during a bacteriological investigation of the same birds, we found that only 4 circovirus, positive velvet scoters were co-infected with *Campylobacter* spp. (3 *Campylobacter jejuni* and 1 *Capmylobacter lari*) while these bacteria were never isolated from any of the tested circovirus, negative velvet scoter (Krawiec M., data not published). Some authors have reported that other bacterial infections, such as *Chlamydia psitaci* and *Salmonella* spp. seem to be more prevalent in birds co-infected with circoviruses [19, 20].

In this study, the circovirus sequence was not detected in other tested bird species. This could be either due to the lack of circoviruses circulating in those species, seasonal factors or the total number of birds from each tested species. For instance, 8 other *Anseriformes* were negative and the mallard duck often harboured the duck circovirus sequence [15, 16].

Based on sequence analysis, the new sequence is related to the duck circovirus. According to *Circoviridae* taxonomy rules, the new circovirus sequence must possess more than 20 % sequence divergence from the already-known viral genome, to be considered as an individual virus species [21]. Therefore, the new circovirus sequence is not divergent enough to be called a distinct virus species, at least when only the DNA sequence is compared.

Based on phylogenetic analysis, it has been proposed that duck circoviruses are grouped into two genotypes DuCV 1 and DuCV 2 [8, 9]. The ICTV does not recognise this division. However, for the phylogenetic analysis of DuCV VS, the distinction of two separate genotypes was considered.

The phylogenetic analysis of the whole genome and the *cap* gene alone, showed that the new circovirus is related to DuCV genotype 1. But analysing the *rep* gene alone gives a different result, suggesting that the new circovirus does not belong to any particular genotype. Detailed phylogenetic analyses of the PCV1 and PCV2, which are the most studied viruses of the *Circoviridae*, revealed that the *rep* gene is subject to the highest selective pressure, and that the *cap* gene should be used for the phylogenetic analysis, if the whole genome sequence is not available [22].

Data presented in this study suggests that the circovirus sequence detected in the velvet scoter belong to the DuCV species and seems to be more related to DuCV 1. Overall, the phylogenetic analysis performed with the new sequence of DuCV VS suggests great diversity among DuCV, as a species.

The velvet scoter and mallard duck belong to distinct sub-families, *Merginae* and *Anatinae*, respectively. It is not known if the circoviruses can cross species barriers and to establish this, the more sophisticated molecular clock analysis should be conducted [23]. As circoviruses are causing loses in domestic duck and geese, there is also a need to obtain more data from wild species of *Anseriformes*.

## Conclusions

The new circovirus sequence belongs to DuCV and was detected in the velvet scoter. Also, on the basis of the relationship of DuCV VS with the duck circovirus, the possibility of cross-infection occurring between farm and free-living birds cannot be discounted. The sequence of the new circovirus found in the velvet scoter suggests that duck circoviruses are very diverse.

## Materials and methods

### Samples

Samples were collected from September 2011 to August 2013. This study included 83 wild birds, from 10 orders and 23 different species, which are presented in Table 1. All tested birds were dead and material, including liver and spleen, were collected during necropsy. Mallard ducks and Eurasian coots were obtained during the hunting season, by two hunting associations, in accordance with local hunting laws, special permissions and hunting programs. Great cormorants were also included and came from the lakes of the Lower Silesia region. Samples from the great cormorants were obtained during the annual population cull, in Poland, with the consent of the Regional Directorate of Environmental Protection, in Wrocław, Poland (no. WPN. 6205.67.2012.MK.1). Samples from velvet scoters, long-tailed ducks, common scoters, razorbills, guillemots, red throated-loons and great crested grebes were collected from birds found dead in fishing nets, off the Baltic coast, between February and April 2013. When found dead, small garden birds were also brought to our University by ornithologists, veterinarians or private persons. The remaining tested, wild bird species were delivered after death, from wildlife rescue centres, where they died before treatment procedures could be introduced. Research was conducted with the consent of the 2nd Local Ethical Committee for Animal Experiments (Wrocław, Poland; no. 41/2011).

During the necropsy of birds, spleen and liver samples were collected and stored at −20 °C, until further processing. Approximately 25 mg of tissue was subjected to DNA isolation.

### DNA extraction, PCR and sequencing

Total DNA was isolated, with a Syngen Tissue DNA Mini Kit (Syngen Biotech, Wrocław, Poland) according to manufacturer’s instructions. During isolation, the sample was treated with 20 μg/ml of RNase A (ThermoScientific). The extracted DNA was eluted in 100 μl of a TE (Tris-hydrochloride buffer, 1 mM EDTA, pH 8.0) and stored at −20 °C. DNA concentration was measured with spectrophotometry and there was approximately 200 ng/ μl of each sample. To amplify the circovirus sequence, the nested PCR, described earlier, was performed [14]. The 25 μ l reaction mixture contained: 0.5 μl of the template DNA, 1× U DreamTaq polimerase (Thermo Scientific) 1× PCR Dream Taq buffer, 80 μM dNTP (Thermo Scientific) and 25 pmol of each of the primers Cv-s (5′AGAGGTGGGTCTTCACNHTBAAYAA3′) Cv-as (5′ AAG GCA GCC ACC CRT ARA ART CRT C 3′) Cn-s (5′ AGC AAG GAA CCC CTC AYY TBC ARG G 3′) and Cn-as (5′ ACG ATG ACT TCN GTC TTS MAR TCA CG 3′) (Genomed, Warszawa, Poland) for the first and second round of PCR, respectively. The reaction mixtures were subjected to a cycling condition, as described in the original publication. The positive control was DNA extracted from the blood of a PBFDV, positive parrot [24]. The second PCR reaction products were visualized on 1.8 % agarose gel, bands were excised from the gel and purified with a Gel-Out kit, according to manufacturer’s instructions (A&A Biotechnology, Gdańsk, Poland). Sanger sequencing was performed with a forward and reverse primer, used for PCR reaction (Genomed, Warszawa, Poland). Readings from both directions were collated and the sequence was subjected to BLAST.

The nucleotide DNA from the positive samples was subjected to another PCR reaction, with primers specific to the duck circovirus *rep* gene, with the primers DuCv forward (5′ CGG CGC TTG TAC TCC GTA CTC 3′ and DuCv reverse (5′ CCC GCG TGG TTT GTA ATA CTT G 3′) [9]. Products of approximately 600 bp were visualised on 1.8 % agarose gel, excised from the gel and purified with a Gel-Out kit, according to manufacturer’s instructions (A&A Biotechnology, Gdańsk, Poland). Sanger sequencing was performed with a forward and reverse primer, used for PCR reaction (Genomed, Warszawa, Poland). Readings from both directions were collated and, using the nucleotide sequence, a new set of primers was designed for amplification of the remaining, circovirus genomic sequence. These primers were as follows: DuCVCloneFor: 5′ CTA GAA CGG CTT CGT CAC CTG ATC 3′ and DuCVCloneRev: 5′ GAA TCC CTG AAG TGT AGG TGT AC 3′. PCR conditions were as follows: 1 cycle at 94 **°**C for 5mins, 35 cycles at 94 **°**C for 30 s, 56 **°**C for 30 s, 72 **°**C for 90 s and 1 cycle of 72 **°**C for 10mins.

For sequencing of the remaining part of the circovirus genome, the PCR reaction was performed on a DNA sample from the spleen of one particular velvet scoter. High fidelity polymerase, Phusion (Thermo Scientific) was used, the conditions were as follows: 1 cycle at 98 **°**C for 5mins, 35 cycles at 98 **°**C for 30s, 60 **°**C for 30s, 72 **°**C for 90s and 1 cycle at 72 **°**C for 10mins. The PCR product was prepared for sequencing, as described above. Primers DuCVCloneFor and DuCVCloneRev were used for sequencing, from both directions. The obtained sequences were aligned to one contig, with SeqMan Pro software (Lasergene, USA). To improve sequencing genome coverage, new primers were designed, with the primer walking method: VS500F (5′AGC GGG TCT AAA TTT ATC AAC AT 3′) VS500R (5′ GCT ATC CTC GCC GGT CAG TCG 3′) VS1100F (5′ CGC CAT TGC CGT TGC TAT CC 3′) and VS1100R (5′ GCT TGT GCC CTC CCG TTC C 3′) which amplified 500 bp and 1.100 bp fragments of the circovirus genome, respectively (cycling condition upon request). The PCR product was sequenced, as mentioned earlier and a new consensus sequence was assembled, from contigs obtained from all previous sequencing results, with the use of SeqMan Pro software (Lasergene, USA). The consensus sequence was checked for circularity, with pairwise alignment (NCBI) of the forward and reverse sequence.

### Sequence analysis

Identification of the putative ORFs was made with the aid of an ORF Finder (NCBI). Sequence analyses were performed with BLAST (NCBI). Nucleotide sequences were aligned and compared to other *Circoviridae* sequences, extracted from GenBank, using the ClustalW algorithm, within MEGA 6.06 software [25]. Phylogenetic analyses were carried out with MEGA 6.06, using the maximum likelihood method and the neighbour joining method, with a bootstrap confidence of 1.000. Amino acid analysis was conducted with ClustaW multiple alignment (NCBI).
